# Inference of the Life Cycle of Environmental Phages from Genomic Signature Distances to Their Hosts

**DOI:** 10.3390/v15051196

**Published:** 2023-05-19

**Authors:** Vicente Arnau, Wladimiro Díaz-Villanueva, Jorge Mifsut Benet, Paula Villasante, Beatriz Beamud, Paula Mompó, Rafael Sanjuan, Fernando González-Candelas, Pilar Domingo-Calap, Mária Džunková

**Affiliations:** 1 Institute for Integrative Systems Biology, University of Valencia and Consejo Superior de Investigaciones Científicas (CSIC), 46980 Valencia, Spainbeatriz.beamud@uv.es (B.B.); rafael.sanjuan@uv.es (R.S.);; 2 Foundation for the Promotion of Sanitary and Biomedical Research of the Valencian Community (FISABIO), 46020 Valencia, Spain; 3 CIBER in Epidemiology and Public Health (CIBEResp), 28029 Madrid, Spain; 4 Department of Space, Earth and Environment, Chalmers University of Technology, 41296 Gothenburg, Sweden; mifsut@student.chalmers.se; 5 Open University of Cataluña, 08018 Barcelona, Spain

**Keywords:** genomic signatures, bacteriophages, lytic phages, lysogenic phages, single-cell genomics

## Abstract

The environmental impact of uncultured phages is shaped by their preferred life cycle (lytic or lysogenic). However, our ability to predict it is very limited. We aimed to discriminate between lytic and lysogenic phages by comparing the similarity of their genomic signatures to those of their hosts, reflecting their co-evolution. We tested two approaches: (1) similarities of tetramer relative frequencies, (2) alignment-free comparisons based on exact k = 14 oligonucleotide matches. First, we explored 5126 reference bacterial host strains and 284 associated phages and found an approximate threshold for distinguishing lysogenic and lytic phages using both oligonucleotide-based methods. The analysis of 6482 plasmids revealed the potential for horizontal gene transfer between different host genera and, in some cases, distant bacterial taxa. Subsequently, we experimentally analyzed combinations of 138 *Klebsiella pneumoniae* strains and their 41 phages and found that the phages with the largest number of interactions with these strains in the laboratory had the shortest genomic distances to *K. pneumoniae*. We then applied our methods to 24 single-cells from a hot spring biofilm containing 41 uncultured phage–host pairs, and the results were compatible with the lysogenic life cycle of phages detected in this environment. In conclusion, oligonucleotide-based genome analysis methods can be used for predictions of (1) life cycles of environmental phages, (2) phages with the broadest host range in culture collections, and (3) potential horizontal gene transfer by plasmids.

## 1. Introduction

During the last decade, metagenomic sequencing has revealed a tremendous quantity of uncultured bacteria, which has also led to the discovery of novel bacteriophages (or phages), viruses of bacteria, making us shift from the laboratory-based discoveries of phages towards the culture-independent identification of phages in metagenomic sequences [1,2]. This has resulted in an enormous expansion of the phage reference databases. Nevertheless, it is already obvious that our ability to gather knowledge on phage biology is not catching up to speed with the sequence-based discoveries.

Phages are the most abundant and variable biological entities and play important roles in global ecosystems, modulating bacterial community composition and mediating horizontal gene transfer [3]. The identification of their hosts and the elucidation of their life cycle are fundamental for assessing their environmental impact [4]. Traditionally, the host range has been evaluated by testing phages against cultures of different host strains, which results in the determination of their preferred life cycle. Phages can either integrate into the bacterial genome and replicate along with the host genome as prophages (temperate phages, lysogenic life cycle), or can form virion particles which are released from the cell, causing cell death (lytic life cycle). Phages can also switch between these two life cycles depending on the growth conditions of the hosts, and some phage families, e.g., *Inoviridae*, release progeny without killing the host cell [3]. Laboratory studies of cultured phages have yielded a list of genes typically associated with lytic or lysogenic life cycle, which boosted the development of computational tools for their detection in the genomes of uncultured phages [5,6,7]. However, most phage genes are poorly conserved; thus, partial matches to these genes do not guarantee their functionality [8]. Phage genomes contain a tremendous variety of genes with unknown functions; most of them are totally unique [9], which suggests that novel types of genes related to lysis or lysogeny are likely to be discovered. Nevertheless, real environmental settings often hide unprecedented phage lifestyles. An example is the crAssphage, the most abundant phage in the human gut, which lacks genes for lysogeny and showed lytic behavior in laboratory settings; however, it does not reduce the population size of its *Bacteroidetes* host [10].

A small portion of the bacterial cells in any environment is infected by phages; thus, they can provide evidence for the actual phage–host links for unculturable bacteria [11]. The presence of a phage in a bacterial cell can be confirmed by the MetaHiC method, which involves the fixation of DNA fragments in close proximity in a single cell [12], digital PCR targeting the presence of specific phages in collected single cells [13], or microbial single-cell genomics, which involves applying phage mining bioinformatic tools to the genome assemblies of single cells [14]. In a previous study, we applied the single-cell genomics approach to reveal the presence of viral contigs in bacterial cells collected from a hot spring microbial mat formed mostly by previously unknown bacteria [15]. This approach allowed us to detect phage–host relationships that could not be discovered by computational tools nor culture-based methods. Then, we analyzed the genome coverage of the detected phages compared to their associated hosts in metagenomes from the same mat layer and adjacent layers, aiming to assess the life cycle of the detected uncultured phages. For most pairs, we detected a close to 1:1 genome coverage ratio, which suggested that phages from these low-mobility environments with high microbial abundance maintain peaceful relationships with their bacterial hosts. Nevertheless, the prediction of the phage life cycle of uncultured phages from metagenomic and single-cell genomic data is limited to studies in which temporal or spatial sample series are available. Therefore, there is a need to develop computational methods for predicting the preferred life cycle of phages from their genome sequences.

Our present study focuses on predicting the preferred lifestyle of phages from their genomic signatures and those of their hosts. Genomic signatures are defined as frequencies of oligonucleotides, and these measures have multiple applications in comparative genomics and evolutionary biology [16]. In 2010, Deschavanne et al. published a genome signature distance approach for predicting the phage life cycle [17], which was based on the “amelioration” hypothesis. This hypothesis posits that the genes acquired by horizontal gene transfer evolve to match the molecular characteristics of the host genome [18] and, consequently, predicts that viral tetranucleotide frequencies are similar to those of their hosts [19]. They investigated a set of 189 cultured phages of *Escherichia coli, Pseudomonas aeruginosa, Staphylococcus aureus*, and *Mycobacterium smegmatis* and observed that phages with a lysogenic life cycle have a shorter genomic signature distance from their host than the lytic phages of the same host.

The method for distinguishing lytic and lysogenic phages proposed by Deschavanne et al. (2010) is based on similarities of tetramer frequencies in the genomes of the phages and their hosts. Longer k-mers could not be used for this purpose, because they result in large data structures (e.g., k = 15 results in a data table with a size of 4 GB) containing a large number of frequencies equal to zero, which hampers comparing genome pairs. Nevertheless, long k-mers can be used for a different approach—the alignment-free comparisons of genomes, which reveal similarities at the genome level without the need for linear alignments or the presence of homologous sequences [20]. For this reason, this approach might be useful for detecting cases of horizontal gene transfer, which is mediated by phages and plasmids [21]. The genomic distances between phages and their hosts obtained by alignment-free comparison do not necessarily correlate with the genomic distances obtained by oligonucleotide frequencies, because two genomes with similar oligonucleotide frequencies do not necessarily have sequences of longer k-mers in common. Therefore, we aimed to explore whether the alignment-free comparison based on matches of longer k-mers reflects the lifestyle of the inferred phages. We hypothesized that there is a universal genomic signature distance threshold which would distinguish phages with lytic and lysogenic life cycles with high precision.

Initially, we obtained a set of bacterial reference genomes with their associated phages in order to: (i) use Deschavanne’s method [17] for calculating genomic distances between bacterial species and their phages based on tetranucleotide frequencies and extend the reference dataset to the bacterial strain level; (ii) calculate genomic distances of inquired bacterial strains to phages not associated with them; (iii) calculate genomic distances of inquired bacterial strains to plasmids that are directly associated or not to these strains. We applied the same methods for phages and plasmids because of the reduced capability of computational tools for distinguishing between phage-like and plasmid-like contigs in metagenomic assemblies of environmental microbiomes. In order to test the resolution of the distance threshold determined from our NCBI reference genome dataset at the strain level, we used a large set of Klebsiella pneumoniae genomes and their phages, which were tested in our laboratory to assess their lytic interactions [22]. Knowing that phages can switch their life cycles between lytic and lysogenic and evolve to adapt to their new hosts in a new environment [23], we decided to apply our computational methods to samples from natural environments that are known to contain a large portion of uncultured microbes [24]. We used the dataset from our previous hot spring microbial mat study [15], containing phage–host links obtained by single-cell genomics and information about their lifestyle assessed from a series of related metagenomic samples. The last objective of our study was to analyze the bacterial and phages genomes by a new alignment-free method based on exact matches of longer oligonucleotides and compare it to Deschavanne’s method [17].

## 2. Results

### 2.1. Set of Reference Genomes Used for Predicting Phage Life-Cycles

For preparing the set of reference bacterial genomes and their phages and plasmids, we considered all strains of bacterial genera with at least five lytic- and five lysogenic-associated phages available in NCBI Virus RefSeq. This resulted in a collection of 5186 bacterial genomes belonging to the genera *Escherichia* (n = 2342), *Lactococcus* (n = 75), *Listeria* (n = 343), *Pseudomonas* (n = 727), *Salmonella* (n = 71), *Shigella* (n = 111), *Staphylococcus* (n = 918), and *Vibrio* (n = 539), which were associated with 284 phages and 6482 plasmids in total (Supplementary Tables S1–S3). The first step was to modify the approach developed by Deschavanne et al. (2010) for distinguishing lytic and lysogenic phages by calculating the genomic signature distances (Euclidian distances) based on similarities of tetramer frequencies (k4freq). While Deschavanne et al. (2010) used only one representative bacterial genome for each bacterial species tested, we extended this set to all available strains of the host genera in NCBI RefSeq, aiming to analyze the reactivity of phages with different bacterial strains [25]. We worked with all strains belonging to the included bacterial genera, because most phage genomes deposited in the NCBI Virus RefSeq database are associated with their host only at the genus level, and the strain- or species-level resolution is not provided.

The methods described next were developed in our study for their application to metagenomic samples, in which the phage host range is not known a priori. We tested the uniformity of the genomic content of all strains in our NCBI reference genome set. Principal component analysis (PCA) based on the k = six genomic signatures of the downloaded bacterial genomes (see Methods) showed a separation of the reference genomes from eight bacterial genera into nine clusters (Supplementary Figure S1, Supplementary Table S1): *Salmonella* (*Salmonella* n = 71), *Escherichia/Shigella* (*Escherichia* n = 2342, *Shigella* n = 111), *Listeria* (*Lactococcus* n = 5, *Listeria* n = 343), *Vibrio1* (*Vibrio* n = 228), *Vibrio2* (*Vibrio* n = 311), *Pseudomonas1* (*Pseudomonas* n = 498), *Pseudomonas2* (*Pseudomonas* n = 229), *Staphylococcus* (*Staphylococcus* n = 918), *Lactococcus* (*Lactococcus* n = 70). Merging genomes classified as *Escherichia* and *Shigella* into one hexamer-based cluster is not surprising, since there is increasing phylogenetic evidence demonstrating the need for the reclassification of these similar taxa [26]. The genera *Pseudomonas* and *Vibrio* were split into two groups, which is a consequence of their diverse genetic composition, allowing it to colonize various environments [27,28]. The PCA also identified 60 genomes that did not form part of any cluster (Supplementary Figure S1), probably belonging to distant species with an insufficient number of representatives to form a cluster, which were removed from our analysis. In summary, the clustering based on hexamer frequencies allowed us to obtain clusters with a similar genomic content, independent of their taxonomic classification.

### 2.2. Distinguishing between Lytic and Lysogenic Phages from the Reference Dataset by the k4freq Method

**Figure 1:**
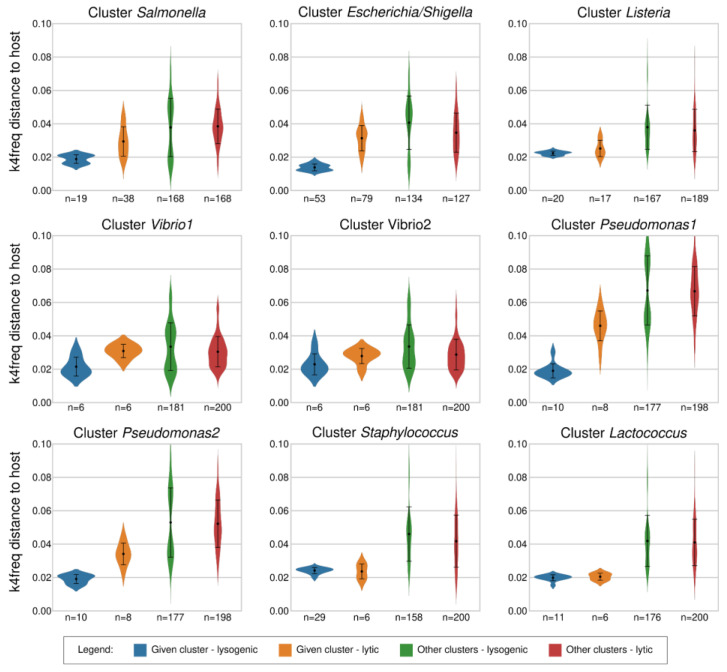
k4freq-based distances of the strains belonging to nine bacterial clusters to their own lysogenic phages, their own lytic phages, and the lysogenic and lytic phages associated with bacteria from other clusters. The figure shows larger differences between lytic and lysogenic phages of *Pseudomonadota* (*Escherichia*, *Pseudomonas*, *Salmonella*, *Shigella*, *Vibrio*) than between those of *Bacillota* (*Lactococcus*, *Listeria*, *Staphylococcus*).

Our first objective was to distinguish between lytic and lysogenic (temperate) phages by analyzing genomic distances of the reference phages to the resulting bacterial clusters as well as to the original genus-level bacterial groups (respecting the NCBI taxonomic classification). Our test set consisted of phages of *Escherichia* (72 lytic, 48 lysogenic), followed by *Salmonella* (38 lytic, 19 lysogenic), *Staphylococcus* (6 lytic, 29 lysogenic), *Listeria* (6 lytic, 8 lysogenic), *Pseudomonas* (8 lytic, 10 lysogenic), *Lactococcus* (6 lytic, 11 lysogenic), *Shigella* (7 lytic, 4 lysogenic), and *Vibrio* (12 lytic, 6 lysogenic). The average distance of the lysogenic phages to their host group, calculated by the k4freq method, ranged from 0.014 ± 0.002 in *Escherichia/Shigella* to 0.024 ± 0.004 in *Staphylococcus* hexamer-based clusters (Figure 1), which were similar to the values obtained by genus-level clustering—ranging from 0.014 ± 0.002 in the *Shigella* genus group to 0.024 ± 0.002 in the *Staphylococcus* genus group (Supplementary Figure S2).

The genomic distances obtained for the lytic phages by the k4freq method ranged from 0.031 ± 0.008 in *Escherichia/Shigella* to 0.046 ± 0.009 in *Pseudomonas1* hexamer-based clusters, and similar values were obtained by genus-level groups (minimum 0.032 ± 0.007 in *Escherichia* and maximum 0.042 ± 0.008 in *Pseudomonas* genus-level groups). In summary, lysogenic phages had significantly shorter k4freq-based genomic distances to their hosts than the lytic phages if the host–phage pairs were analyzed globally across all hexamer-based clusters or genus-level groups (global comparisons in Supplementary Table S4). Nevertheless, the Kruskal–Wallis H-test showed that *Lactococcus*, *Staphylococcus*, and *Vibrio2* clusters did not have statistical differences between their lytic and lysogenic phages (*p*-values 0.55, 0.90, and 0.15, respectively). Altogether, the results indicate that it is easier to differentiate between lytic and lysogenic phages of bacterial genera belonging to the phylum *Pseudomonadota*—formerly *Proteobacteria* (*Escherichia*, *Pseudomonas*, *Salmonella*, *Shigella*, *Vibrio*) than those from the phylum *Bacillota*—formerly *Firmicutes* (*Lactococcus*, *Listeria*, *Staphylococcus*). The average k4freq genomic distances of lytic and lysogenic phages to all strains of their associated hosts were 0.030 ± 0.008 and 0.019 ± 0.005, which sets the threshold for distinguishing the two groups to 0.026 (Supplementary Figure S3).

**Figure 2:**
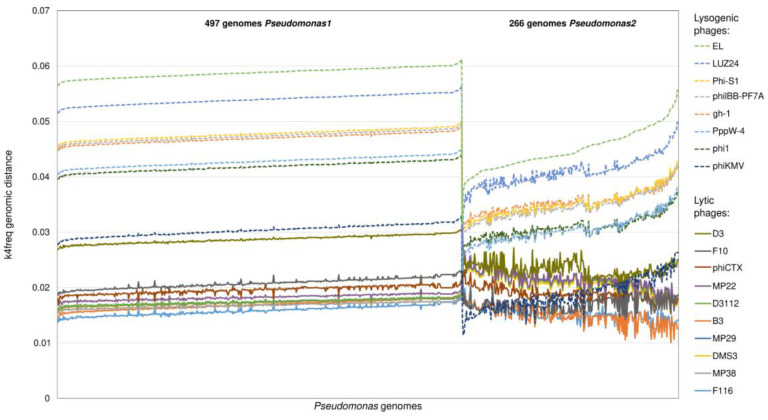
Details of the k4freq-based distances of the two *Pseudomonas* clusters to their own phages.

The clustering of bacterial genomes into hexamer-based groups (Supplementary Figure S1) helped to uncover the different reactivity of phages associated with a single bacterial genus. The differences in the genomic distances for the lytic and lysogenic phages were 1.8× greater in the *Pseudomonas1* cluster than in the *Pseudomonas2* cluster, due to the decrease in the k4freq values of the seven lytic phages when compared to the *Pseudomonas2* strains (Figure 1 and Figure 2). The most striking differences were observed for the *Pseudomonas* phage phiKMV—its k4freq genomic distances to the strains in the *Pseudomonas1* cluster were within the values of the lytic phages; however, these distances decreased in the case of strains belonging to the *Pseudomonas2* cluster reaching the lysogenic area (Figure 2).

We also analyzed the k4freq-based genomic distances between bacterial genomes and phages that are not associated with these bacteria (third and fourth violin plots in Figure 1 and Figure S2). The vast majority of phages that were not associated with the inquired host group had k4freq-based distances that were larger than the average value calculated for the lysogenic phages associated with the inquired host group. Nevertheless, there were no significant differences between the non-associated phages and lytic phages of the inquired bacterial group.

### 2.3. Comparison of k14exact and k4freq Methods

**Figure 3:**
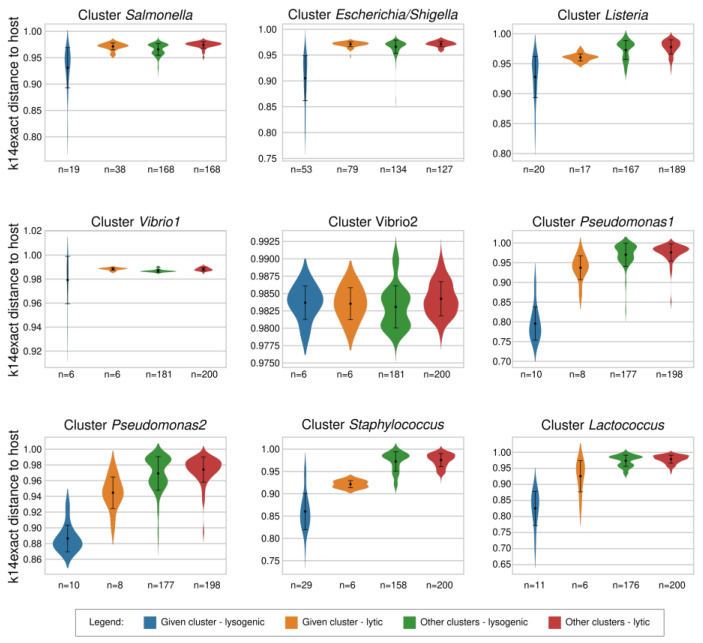
k14exact-based distances of the strains belonging to nine bacterial clusters to their own lysogenic phages, their own lytic phages, and the lysogenic and lytic phages associated with bacteria from other clusters. The figure shows larger differences between lytic and lysogenic phages of *Pseudomonadota* (*Escherichia*, *Pseudomonas*, *Salmonella*, *Shigella*, *Vibrio*) than between those of *Bacillota* (*Lactococcus*, *Listeria*, *Staphylococcus*).

We began by calculating the optimal length of k-mers for the new method for distinguishing lytic and lysogenic phages, for which we had to consider the length of bacterial genomes, aiming to obtain the lowest rate of possible random matches between bacterial and phage genomes. The equations proposed by Swain & Vickers et al. [29] revealed that the k-mers of 14 and 15 nucleotides would result in <2% of random matches. However, the option with k = 15 would require 4x longer computational time (Supplementary Figure S4); thus, we opted for oligonucleotides k = 14 (k14exact). The differences between the average genomic distances of lytic and lysogenic phages were larger with the k14exact than with the k4freq method in all tested groups, except for *Vibrio1* and *Vibrio2* clusters, in which lytic and lysogenic phages had similar genomic distances from their host using both methods (Figure 1 and Figure 3). The average k14exact genomic distances of lytic and lysogenic phages to all strains of their associated hosts were 0.965 ± 0.020 and 0.895 ± 0.059, which sets the threshold for distinguishing the two groups to 0.955 (Supplementary Figure S3). We observed an important advantage of the k14exact method compared to k4freq—it provided better distinguishing between lytic and lysogenic phages for the *Bacillota* phylum. While no statistical differences were detected for lytic and lysogenic phages of *Staphylococcus* with k4freq (Figure 1), the k14exact method (Figure 3 and Figure S5) yielded values with significant differences (*p* = 0.025, Supplementary Table S4). Similar to the k4freq method, the k14exact method yielded no significant differences between lytic phages of the inquired bacterial group and phages not associated with this bacterial group.

### 2.4. Reference Genomes Set Tested for the Strain-Level Association of Plasmids

**Figure 4:**
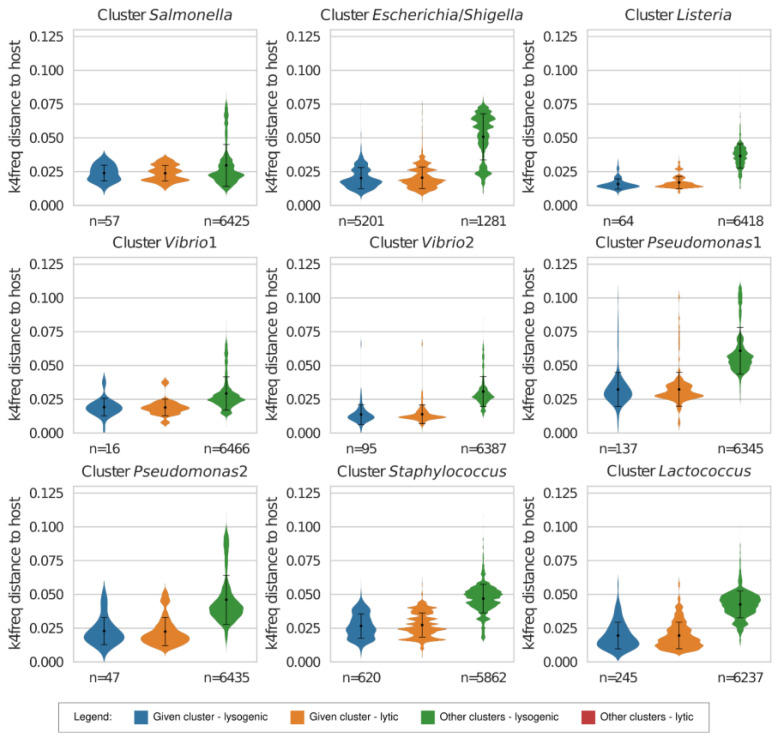
k4freq-based distances of the strains belonging to nine bacterial clusters to their own plasmids, to the plasmids associated with other bacterial genomes from the same cluster, and to the plasmids associated with bacteria from other clusters.

The last step of the exploration of our NCBI genome reference dataset involved calculating k4freq and k14exact distances for bacteria and their associated plasmids and plasmids not associated with them, which, in total, comprised a set of 6482 plasmids (*Escherichia* n = 4963, *Lactococcus* n = 253, *Listeria* n = 56, *Pseudomonas* n = 184, *Salmonella* n = 57, *Shigella* n = 238, *Staphylococcus* n = 620, and *Vibrio* n = 111). In contrast to the phages in the NCBI Virus RefSeq, plasmids are associated with concrete bacterial strains in this database, although experimental data show that many of them can move between different bacterial strains or species. We expected that each bacterial strain will have the shortest genomic distance to the plasmid(s) precisely associated with it. Nevertheless, the results of the k4freq analysis revealed no significant differences between the plasmids associated with the inquired strain (0.021 ± 0.009) and the plasmids associated with the other strains belonging to the same hexamer-based cluster (0.021 ± 0.008, Figure 4 and Figure S4).

**Figure 5:**
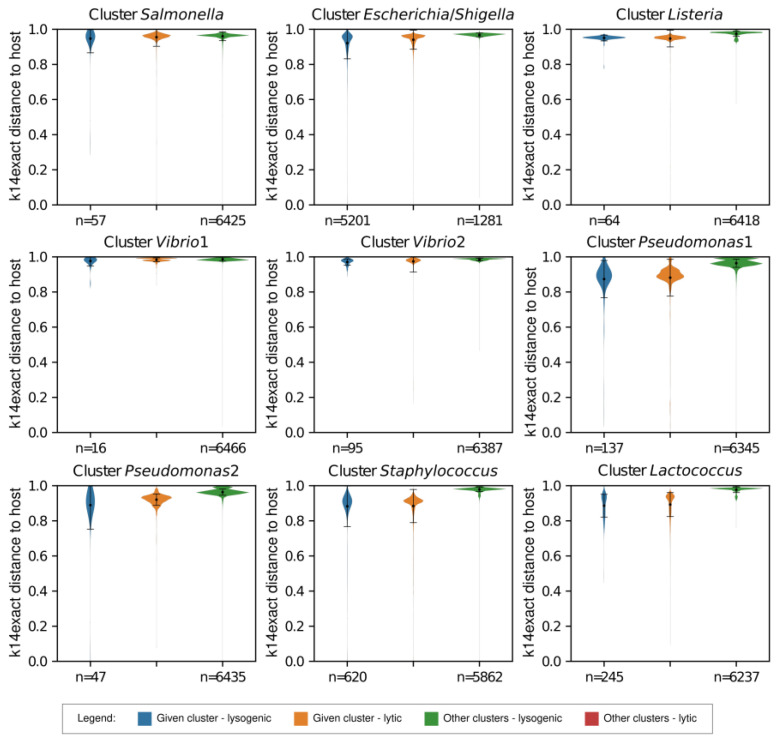
k14exact-based distances of the strains belonging to nine bacterial clusters to their own plasmids, to the plasmids associated with other bacterial genomes from the same cluster, and to the plasmids associated with bacteria from other clusters.

The same was observed for the k14exact distances: the genomic distances of the associated plasmids (0.938 ± 0.058) did not differ from the distances of the plasmids associated with the other strains from the same bacterial group (0.938 ± 0.058, Figure 5). The group of plasmids associated with bacteria from other hexamer-based clusters had significantly larger k4freq (0.045 ± 0.017, Figure 4) and k14exact (0.974 ± 0.017, Figure 5) distances than the plasmids associated with the inquired bacterial group (with *p* < 0.0001). The k14exact method proved to be very useful for the detection of plasmids that share a large portion of their genetic information with most of the genomes in our reference datasets, including distant taxa (visualized as outliers in Figure 5, e.g., *Vibrio1*).

### 2.5. Interactions of Klebsiella Pneumoniae Strains with Their Lytic Phages

The genomic distance values obtained for all strains within their hexamer-based clusters suggested that the k4freq and k14exact methods do not provide strain-level resolution of their association with the phages. In order to explore the strain-level resolution of the k4freq and k14exact methods, we included an additional experimental dataset, because the NCBI Virus RefSeq does not provide strain-level resolution of the phages–host interactions. We analyzed a set of 5658 combinations of 41 *Klebsiella* phages and 138 *Klebsiella* strains that had been tested in our laboratory for their interactions [22]. This set contained phages that were capable of infecting from 1 to 17 *Klebsiella* strains. The strains had different levels of resistance to the tested phages: there were 76 *Klebsiella* strains that resisted all tested phages, while the remaining 62 strains were lysed by at least one phage and maximally by nine phages from this collection. The genome analysis of these phages in our previous study showed that they belong to 13 genomic similarity groups. However, phages from the same genomic group did not react with the same *Klebsiella* strains. The k4freq and k14exact showed that phages from the same genomic group had similar genomic distances to the *Klebsiella* strains, which means that these genomic distances do not provide strain-level resolution of the phage specificity (Supplementary Figure S6).

**Figure 6:**
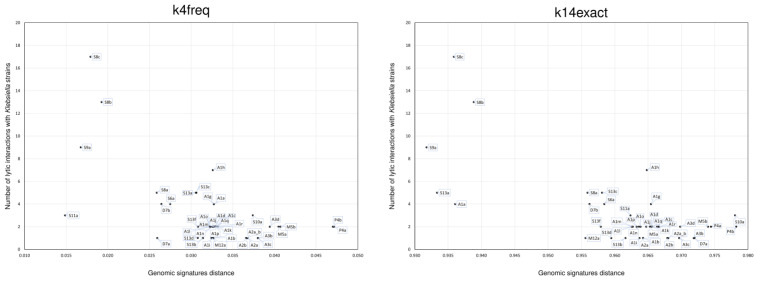
Average of k4freq and k14exact distances of the phages to all *Klebsiella* strains and the number of their lytic interactions detected in the laboratory.

Nevertheless, our results showed that the genomic distances can identify phages with the highest number of lytic interactions with different strains of the same species. The lytic lifestyle of the *Klebsiella* phages was confirmed in the present study by the average values obtained for k4freq (0.032 ± 0.007) and k14exact (0.962 ± 0.011) genomic distances, which fell within the range calculated for the lytic phages using the NCBI reference dataset (Figure 6 and Figure S7).

Interestingly, the *Klebsiella* phages showing the largest number of lytic interactions had the shortest genomic distances to *Klebsiella*: four phages (S8b, S8c, S9a, S11a) were below the lytic threshold established for k4freq (0.026), and five phages (A1a, S8c, S8b, S9a, S13a) were below the lytic threshold established for k14exact (0.955). The phages with more lytic interactions tended to have short k4free and k14exact distances, which indicates that both methods can be used for the identification of highly reactive lytic phages (Figure 6). Interestingly, these highly reactive lytic phages in the “lysogenic area” were in fact later experimentally shown to have a lysogenic relation with other *Klebsiella* strains (Supplementary Table S5). This shows that it is very difficult to draw a clear line to separate phages with lytic and lysogenic lifestyles, which clearly depend on the host strain. These difficulties were reflected in the disagreements of the phage lifestyle predictions made by the four bioinformatic approaches (PhageAI, PHACTS, BACPHLIP, presence of genes associated with lysogeny) used in our study (Supplementary Table S5). The genomic distance methods developed in our study represent yet another bioinformatic approach, which in some cases was more effective than the previously available tools. For example, the other four bioinformatic tools indicated that the phage A1a was lytic; however, it was detected in the “lysogenic area” by the k14exact method, and, in fact, its lysogenic activity was observed in the laboratory with the *K. pneumoniae* strain 852 (Supplementary Table S5).

### 2.6. Host–Phage Interactions Predicted from Bacterial Single Cells

The knowledge on phage–bacteria genomic distances, gathered when testing the NCBI reference genomes set and *Klebsiella* phages, was applied to a phage–host interaction matrix obtained from single-cell genomics data from our previous study on a hot spring microbial mat (Jarrett et al., 2020). We analyzed a set of 24 diverse bacterial species belonging to the phyla *Acidobacteria* (n = 4), *Armatimonadetes* (n = 1), *Bacteroidetes* (n = 4), *Desulfobacteraeota* (n = 4), *Nitrospirae* (n = 1), *Patescibacteria* (n = 2), *Planctomycetes* (n = 1), *Pseudomonadota* (n = 1), *Spirochaetes* (n = 2), *Verrucomicrobia* (n = 2), and *Zixibacteria* (n = 1). We calculated k4freq and k14exact distances between these 24 bacterial genomes and 41 phages detected in single-cell assemblies (Supplementary Table S6), indicating that these phages were located either inside the cells of their bacterial hosts or attached to the cell surface. In our previous study, the analysis of their genome coverages in metagenomic samples from different layers of the same microbial mat suggested that these phages had a temperate lifestyle, and in the present study, we aimed to verify it by the k4freq and k14exact methods.

**Figure 7:**
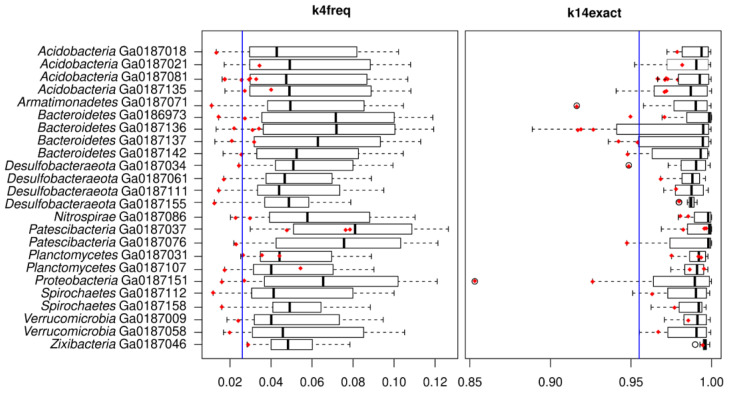
k4freq and k14exact distances of phages and bacteria detected in a hot spring microbial mat by single-cell genomics represented by boxplots showing the middle 50% interquartile range. The red dots represent the viral-like contigs detected in the given single-cell assembly. The blue line corresponds to the thresholds for distinguishing lytic and lysogenic phages calculated using cultured bacteria.

The average genomic distance of the phages to their single cell-associated hosts was 0.028 ± 0.015 for k4freq and 0.965 ± 0.032 for the k14exact method (Figure 7), which was slightly above the lytic/lysogenic threshold established using the NCBI dataset of eight cultured *Bacillota* and *Pseudomonadota* genera described above (0.026 for k4freq and 0.955 for k14exact).

Nevertheless, these values were still lower than the maximum genomic distances of the lysogenic phages to their associated hosts detected in the NCBI dataset, which means that the obtained genomic distances do not exclude the lysogenic lifestyle of these phages. The genomic distances of the 24 bacterial genomes to the non-associated phages from the same microbial mat (0.059 ± 0.026 for k4freq and 0.987 ± 0.032 for k14exact, Figure 7) were higher than the average of the genomic distances of the NCBI reference dataset bacteria to the non-associated phages from the same dataset (0.042 for k4freq and 0.97 for k14exact). The results from our small single-cell dataset suggest that the genomic distance values calculated for associated and non-associated phages differ more in natural microbiome settings than in our NCBI dataset. Nearly all (>95%) phages had k4freq and k14exact genomic distances to their associated hosts that were shorter than the average of the genomic distances of the same bacterial genomes to the non-associated phages from the same microbial mat (Figure 7). This suggests that the results of the k4freq and k14exact genomic distance calculations are in accordance with the temperate life cycle predicted from the metagenomic spatial series in our previous study.

## 3. Discussion

In the first part of our study, we determined genomic distance thresholds for distinguishing lytic and lysogenic phages using the NCBI reference dataset containing different strains of eight bacterial genera, for which at least five lytic and five lysogenic phages were available. The values of the k4freq-based genomic distances obtained in our study can be compared directly with those from Deschavanne et al. (2010), who also investigated phages of *Escherichia*, *Pseudomonas*, and *Staphylococcus* [17]. They set the k4freq-based genomic distance threshold for distinguishing lytic and lysogenic phages of *Escherichia coli* and *Pseudomonas aeruginosa* to 0.018, but the same threshold cannot be applied to *Staphylococcus*, since some *Podoviridae* lytic phages had genomic distances from the *S. aureus* genome as low as 0.015. The sets of *Staphylococcus* phages used in our study and in the study of Deschavanne et al. (2010) overlapped by two-thirds (differences were due to different criteria for reference genomes selection); however, both studies yielded similar results. Our result slightly differed from the threshold obtained in the study of Deschavanne et al. (2010), in which a smaller dataset was used. Expanding the dataset to an even larger number of taxa would surely result in a higher precision for defining the lytic-lysogenic threshold. Nevertheless, many bacterial genera do not contain a sufficient number of associated lytic and lysogenic phages to perform a proper comparison. The cultivation, experimentation, and reporting biases in the NCBI Virus RefSeq are the reasons why the phages of many well-studied bacterial genera, such as *Streptococcus*, *Klebsiella*, or *Yersinia*, could not be assessed for distinguishing between lytic and lysogenic phages. The lytic-lysogenic threshold might be adjusted in the future, after more knowledge about the lifestyle of uncultured phages is gathered from experimental data on environmental samples (using single-cell genomics and metagenomics). More data on uncultured phages will show whether it is possible to specify a single lytic-lysogenic threshold for all phages or whether the threshold must be adjusted according to the bacterial groups they infect (e.g., different thresholds for different phyla).

Recently developed computational tools for predicting the phage lifestyle based on their genomic signatures, such as PhagePred [30] and DeePhage [31], were designed to function without the need to associate inquired phages with a bacterial host. Nevertheless, these tools have been optimized using the whole NCBI Virus RefSeq database, in which some bacterial groups contain only lytic phages, while others are associated with lysogenic phages only. This suggests that the results generated by these host-free tools might be influenced by the different genomic content of the bacterial hosts of these phages, even if the host genome is not considered. Phages associated with different bacterial groups have very different genomic signatures, which allows for the clusterization of their genomes into groups and the identification of novel phages related to these clusters [32]. In contrast to the above-mentioned host-free computational tools based on genomic signatures, we explored strain-level differences of genomic distances between phages and bacteria. We showed that all the strains from a given hexamer-based cluster had very similar genomic distances to an inquired phage. We did not detect significant differences between genomic distances calculated for the lytic phages associated with the inquired bacterial cluster and the phages not associated with this bacterial cluster (both lytic and lysogenic), which indicates that, in natural communities containing previously unknown phage–bacteria pairs, the true lytic phages of an inquired bacterial group might be confused with lytic or lysogenic phages not associated with that bacterial group. This indicates that before analyzing the phage lifestyle in metagenomic studies, the correct phage–bacteria linkages must be provided at least at the level of host hexamer-based frequencies clusters.

We need to underline that shorter genomic distances between phages and their hosts do not automatically mean that these phages are lysogenic, and they do not exist in a lytic form. Different evolutionary models suggested that there is an evolutionary pressure on lytic phages to switch to lysogeny [33]. Many phages, which are observed in the 0.2 μm filtered fractions of environmental samples as numerous virions, can be detected in the form of prophages integrated into the genomes of bacteria in some of the related samples [34]. Lysogenic phages have the greatest ability to adapt to a variety of bacterial host strains [18], which is evident from the number of highly similar prophages detected across multiple bacterial strains or species [35]. Distant environments are more likely to harbor more reactive phages [23]; therefore, in our previous *Klebsiella* study we aimed to isolate new lytic phages of *K*. *pneumoniae* from environments distant from the human respiratory tract, such as soil. Indeed, we managed to isolate broad host-range phages with lytic interactions with up to 17 *Klebsiella* strains. Surprisingly, these highly reactive phages had the shortest genomic distance from the *Klebsiella* genomes, falling into the “lysogenic genomic distance zone”. The only exception was the S9a phage, which has not been observed in the form of lysogens yet, which points to the reporting and experimental biases mentioned above. Since it seems ambitious to draw a straight line between lytic and lysogenic phages, we suggest that the k4frew and k14exact methods can have another very practical application. For example, they can be used for predicting which phages will have the highest number of lytic interactions with a collection of bacterial host strains. This will considerably reduce the efforts required for testing all the combinations of phages and bacterial strains in the laboratory, aiming to decrease the time necessary for finding phages suitable for personalized phage therapies [36].

Similar to phages, neither the k4freq nor k14exact genomic distance of plasmids to bacterial genomes had strain-level resolution—not all bacterial strains had the shortest distances to the phages associated with them. Nevertheless, we detected significant differences between plasmids associated with the inquired bacterial cluster and plasmids not associated with this cluster, which means that we reached better resolution than the PlasFlow genomic signatures-based computation tool [37] and %GC content-based analysis [38] that provided phylum-level resolution. Neither random forest method employed in the PlasmidHostFinder tool [39] reaches strain-level resolution. Nevertheless, for the analysis of metagenomic samples, linking plasmids to their bacterial host group (species-like group) seems to be sufficient, because the binning of metagenome-assembled genomes (MAGs) rarely provides strain-level resolution [40]. Some plasmids can be acquired by a wide range of bacterial species or genera, which is reflected in their genomic signatures [41]. The detection of wide-range host plasmids is the most important application of the genomic distance analysis; it can be used, for example, for predicting the dissemination of antimicrobial resistance genes on a global scale, showing the potential of the acquisition of plasmids from soil by bacteria from the human microbiome [42]. In our study, the k14exact method was shown to be very efficient in identifying plasmids that share large portions of their DNA sequence with non-associated bacteria. It means that this method identifies plasmids actively contributing to the horizontal gene transfer across bacterial genera.

In conclusion, the genomic distance methods have several practical applications—they can be used for predicting (i) the lifestyle of environmental phages if they are associated with a bacterial host at least on a species-like level, which is important for assessing their environmental impact, (ii) phages with a wide range of host strains, which is useful for the development of personalized phage therapies, (iii) the detection of plasmids with the largest potential for the dissemination of antimicrobial resistance genes across distant bacterial taxa.

## 4. Methods

### 4.1. Datasets

The RefSeq genomes of phages were downloaded from NCBI Virus on 2 March 2022. For the ensuing analyses, we selected only those phages that had information about their life cycle provided by Mavrich & Hatfull [43]. Bacterial host genera were selected if there were at least five lytic and five lysogenic phages associated with them. Complete genomes or genomes at the chromosome assembly level of the associated host genera were downloaded from RefSeq, along with their associated plasmids. In total, we worked with a set of 291 phages, 6482 plasmids, and 5186 bacterial genomes from 8 genera.

In the second analysis, we used a set of genomes of 41 lytic phages isolated in our laboratory on *K. pneumoniae* and 138 *K. pneumoniae* strains, reported in our previous study [22].

The last analysis was performed with pairs of environmental phages and their hosts associated on a single-cell level from our previous study [15].

### 4.2. Genomic Signatures Based on Oligonucloetide Frequencies

For a given k-mer *w*, its occurrence in a contig *X* is defined as $X_{w}$ and the relative frequency of this k-mer is defined as:

$$f_{w}^{X}=\frac{X_{w}}{\sum_{w}X_{w}}$$

From these measurements, a principal component analysis was carried out, and the resulting projection was analyzed using the DBSCAN (Density-based spatial clustering of applications with noise) clustering algorithm. Values of eps = 0.50 and MinPts = 10 in DBSCAN were used in order to obtain a small number of relatively dense clusters.

The value *k* = 6 was chosen for the clustering of bacterial genomes in the NCBI reference set. Afterwards, we calculated the distances of bacterial strains to the phages and plasmids according to Deschavanne et al. (2010), which was adjusted to *k* = 4, taking into the account the shorter length of the plasmids/phage genomes [17].

Following the guidelines of Vinga & Almeida (2003) [44], we calculated the Euclidean distance (k4freq) between the pairs of genomes:

$$Eu\left(X,Y\right)=\sqrt{\sum_{w\in S^{k}}{\left|f_{w}^{X}-f_{w}^{Y}\right|}^{2}}$$

### 4.3. Alignment-Free Genomic Distances

In this study, we proposed a new distance of similarity for high values of *k* (*k* > 14), which is a variation of the distance D2 defined by Swain &Vickers (2022) [29], where the value of 4^k^ is two orders of magnitude larger than the size of the largest genome.

$$SX=\sum_{w}X_{w}$$

$$SY=\sum_{w}Y_{w}$$

If SX<SY, we define the similarity function SIM between two sequences as:

$$SIM\left(X,Y\right)=\frac{\sum_{w}X_{w}}{SX}\forall \left(Y_{w}>0\right)$$

Finally, we define the distance measure *DSW*(k14exact) as the inverse of the Similarity function, as follows:

$$DSW\left(X,Y\right)=\left(1-SIM\left(W,Y\right)\right)$$

The obtained distance is normalized to values between 0 and 1. If the *DSW* = 0, it means that the genome *X* is inserted into the genome *Y*. If *DSW* = 1, there is no similarity between two genomes (they do not share any oligonucleotides of length *k* = 14).

### 4.4. Determination of Thresholds for Distinguishing Lytic and Lysogenic Phages

In order to estimate the performance of each classifier, its corresponding receiver operating characteristic curve (ROC curve) was constructed, and the area under the curve (AUC) was calculated. The best threshold was determined from the point on the curve closest to the ideal classifier, which corresponds to the point (0, 1) in the ROC space.

### 4.5. Determination of Lysogenic Activity

The screening for lysogenic activity was conducted by evaluating 30 phage-resistant colonies for the spontaneous release of phage particles, following the protocol of Gordillo Altamirano & Barr (2021) [45].The preferred lifestyle of the lytic *Klebsiella* phages was determined by the k14exact and k4freq methods, as described above, and by four other bioinformatic approaches: (1) PhageAI [7], (2) PHACTS [5], (3) BACPHLIP [46], (4) looking for the presence of proteins related to lysogenic lifestyle (replication and partition system proteins ParA/B, integrase, excisionase, repressors, and Cro/C1-like proteins) by a semantic search from functional annotation with PHROGS [47].

## Data Availability

Not applicable.

## Supplementary Materials

The following supporting information can be downloaded at: https://www.mdpi.com/article/10.3390/v15051196/s1. Figure S1: PCA of genomic signatures based on hexamer frequencies of the reference bacteria; Figure S2: k4freq-based distances of the bacterial genera to the reference phages; Figure S3: ROC curves for k4freq and k14exact; Figure S4: Statistical error analysis for different k-mer options; Figure S5: k14exact-based distances of the bacterial genera to the reference phages; Figure S6: Distances of 75 *Klebsiella* strains to 41 *Klebsiella* phages; Table S1: Bacterial genomes in the reference set; Table S2: Phage genomes in the reference set; Table S3: Plasmids in the reference set; Table S4: Summary of statistical comparisons of genomic distances; Table S5: Lysogenic activity of *K. pneumoniae* phages; Table S6: Bacterial single-cells and phages detected in their assemblies
