# Supplementary material for: Inference of the Life Cycle of Environmental Phages from Genomic Signature Distances to Their Hosts
Source: Viruses. 2023 May 19;15(5):1196. doi: 10.3390/v15051196 (PMC10222151; doi:10.3390/v15051196)

Supplementary Figures

Inference of the life cycle of environmental phages from genomic signature distances to their hosts

Vicente Arnau, Wladimiro Díaz-Villanueva, Jorge Mifsut Benet, Paula Villasante, Beatriz Beamud, Paula Mompó, Rafael Sanjuan, Fernando González-Candelas, Pilar Domingo-Calap, Mária Džunková

Supplementary Figure S1: PCA of genomic signatures based on hexamer frequencies of the reference bacterial genomes, showing detected genome clusters (panel a) and colored by bacterial genus (panel b).

a

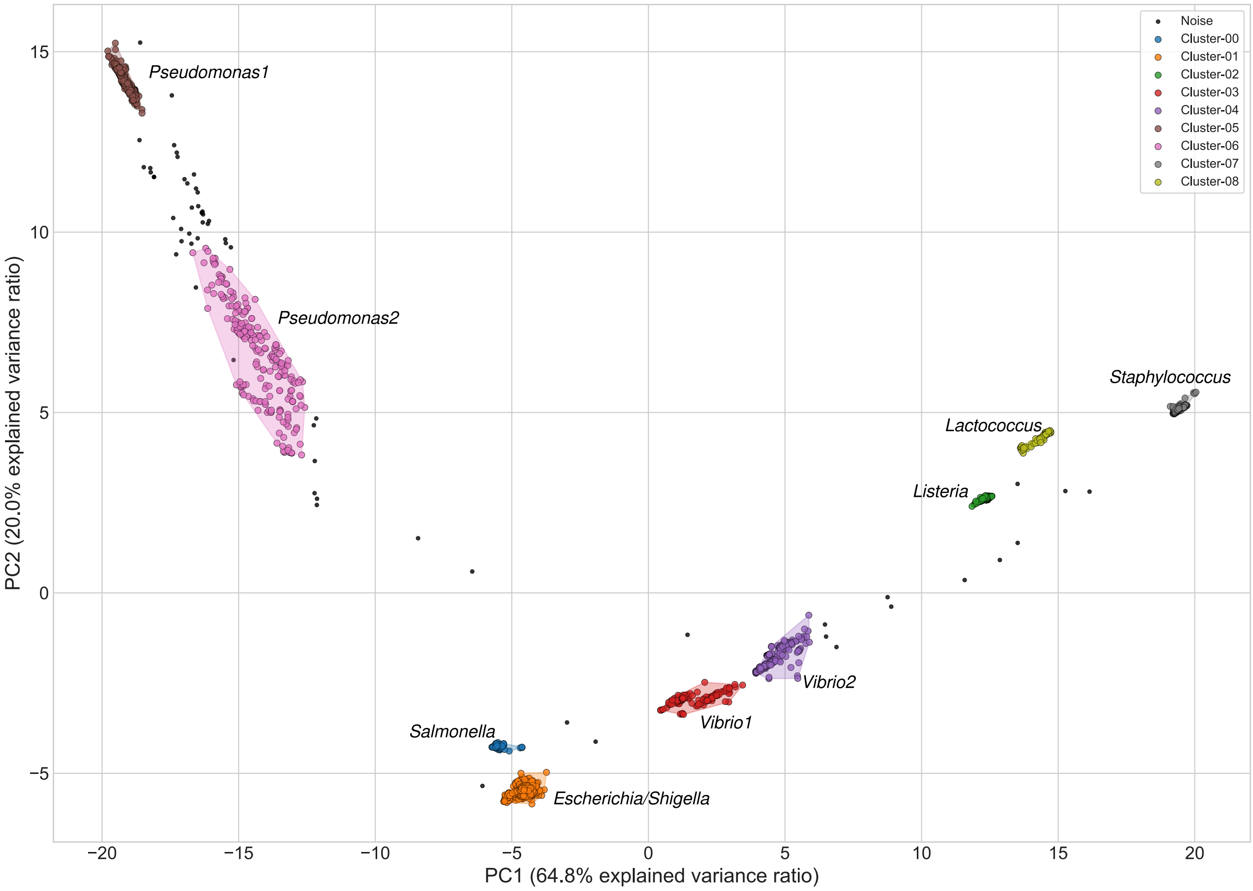

b

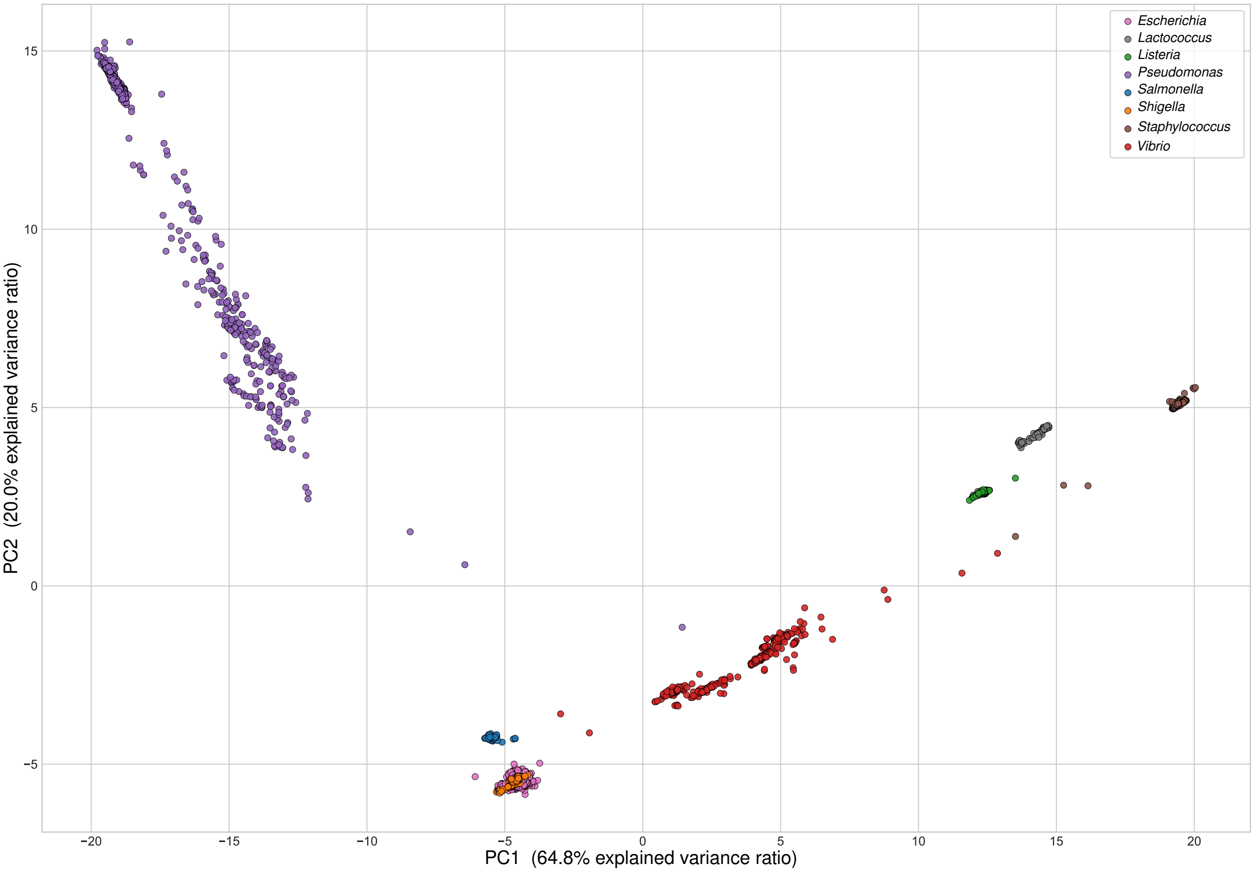

Supplementary Figure S2: k4freq-based distances of the strains belonging to 8 bacterial genera to their own lysogenic phages, their own lytic phages, and the lysogenic and lytic phages associated to bacteria from other genus-based groups.

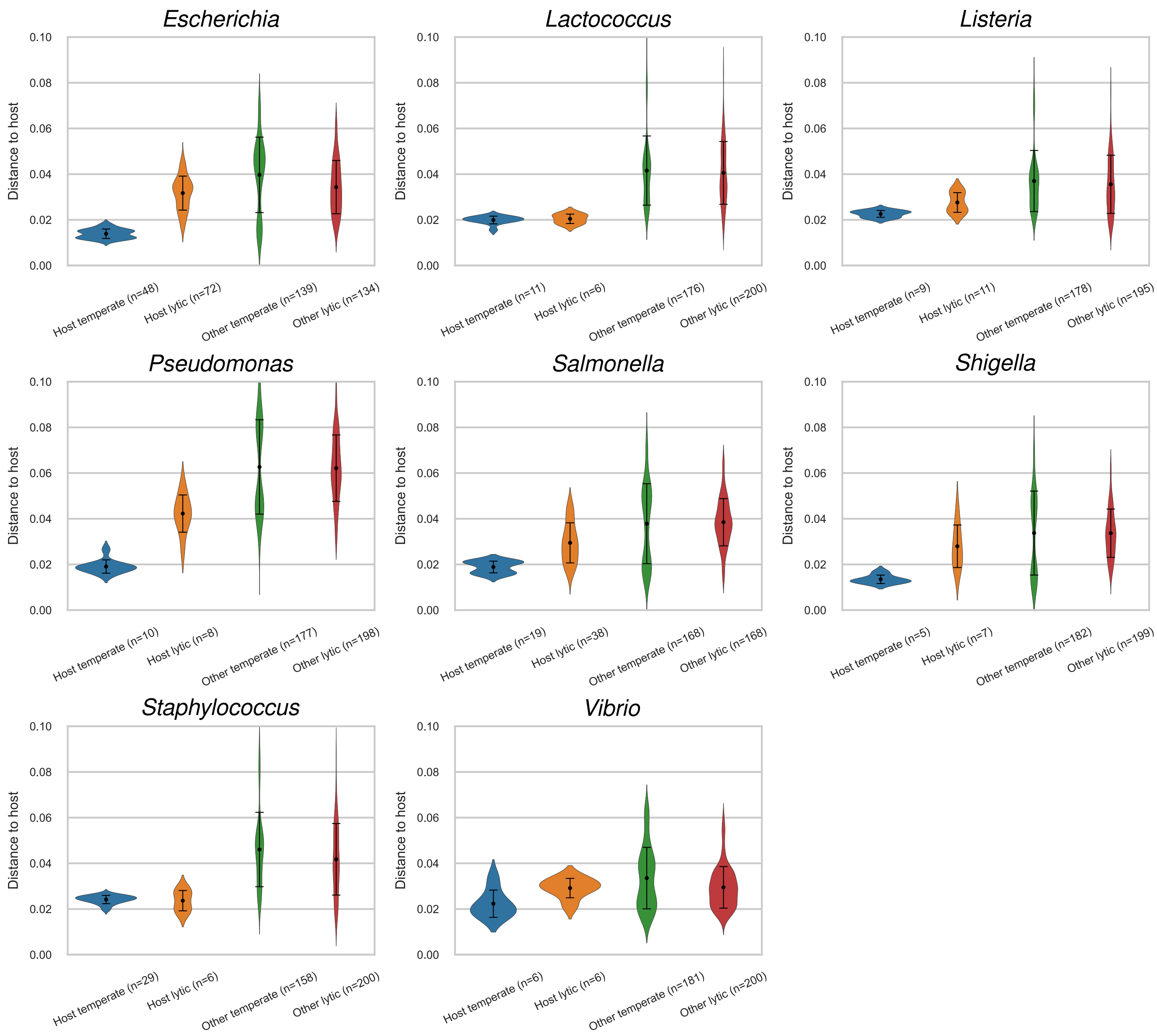

Supplementary Figure S3:

Definition of the threshold for distinguishing lytic and lysogenic phages by receiver operating characteristic curve (ROC curve). The ROC curve is defined as the plot of the true positive rate (TPR) against the false positive rate (FPR) considering the threshold used in the classifier as a parameter. The so-called ROC space is given by all possible results of such a classifier in the form (FPR,TPR). The performance of any classifier (with the corresponding threshold included) can be represented by a point in the ROC space. ROC curves move from the "lysogenic" point (0,0) which corresponds to the lowest value of the threshold to the “lytic” point (1,1) given by the highest value for the threshold. The straight line between these two trivial points in the ROC space corresponds to the family of random classifiers with different a priori probabilities for each class. The more a ROC curve separates from this line, the better the corresponding classification scheme is. As ROC curves move away from this line, they approach the best possible particular result that corresponds to the point (0, 1) in the ROC space. The ROC curve is a perfect tool to find the best trade-off between true positives and false positives and to compare classifiers in a range of different situations. k4freq TPR: 65.5% and FPR: 3.1%, k14exact TPR: 84.5% and FPR: 10.4%.

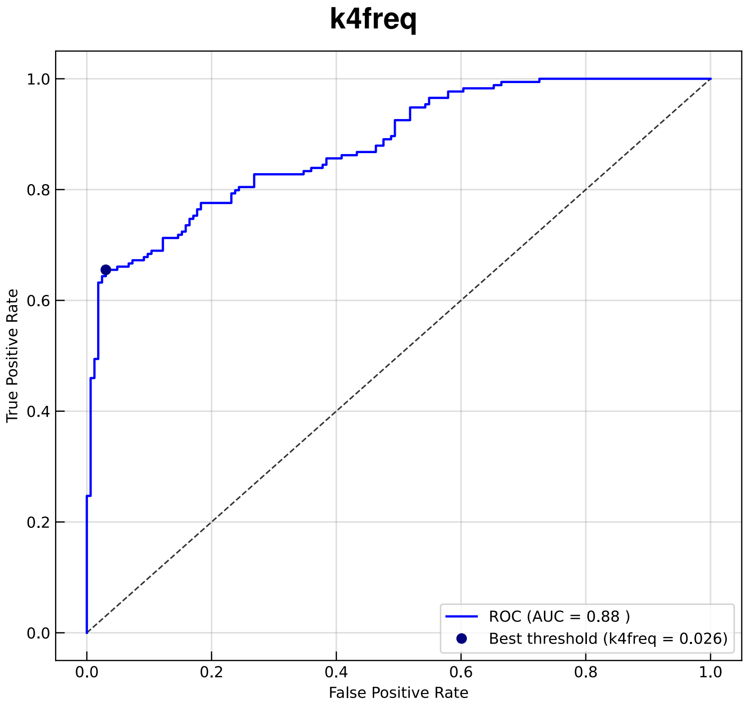

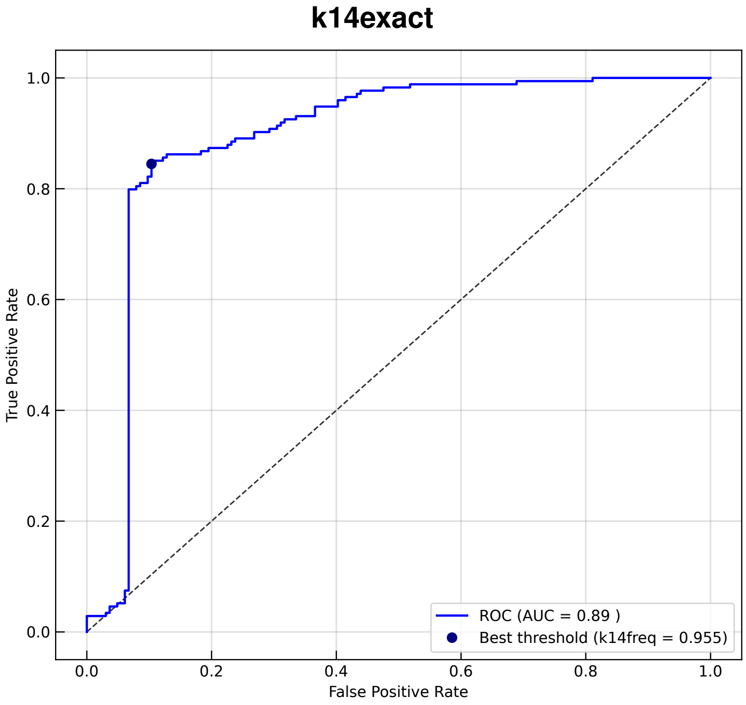

Supplementary Figure S4:
Statistical error analysis for different k-mer options, when applied to different genome sizes

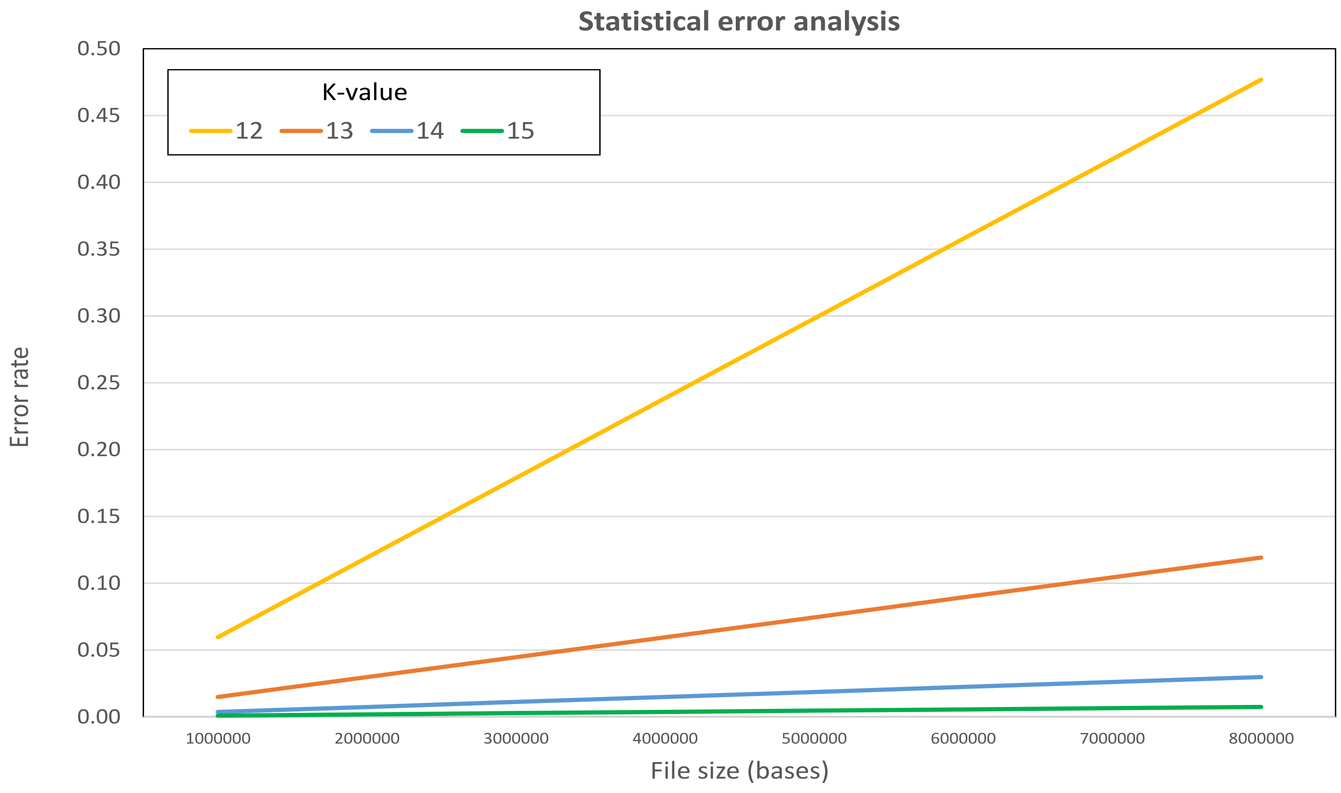

Supplementary Figure S5: k14exact-based distances of the strains belonging to 8 bacterial genera to their own lysogenic phages, their own lytic phages, and the lysogenic and lytic phages associated to bacteria from other genus-based groups.

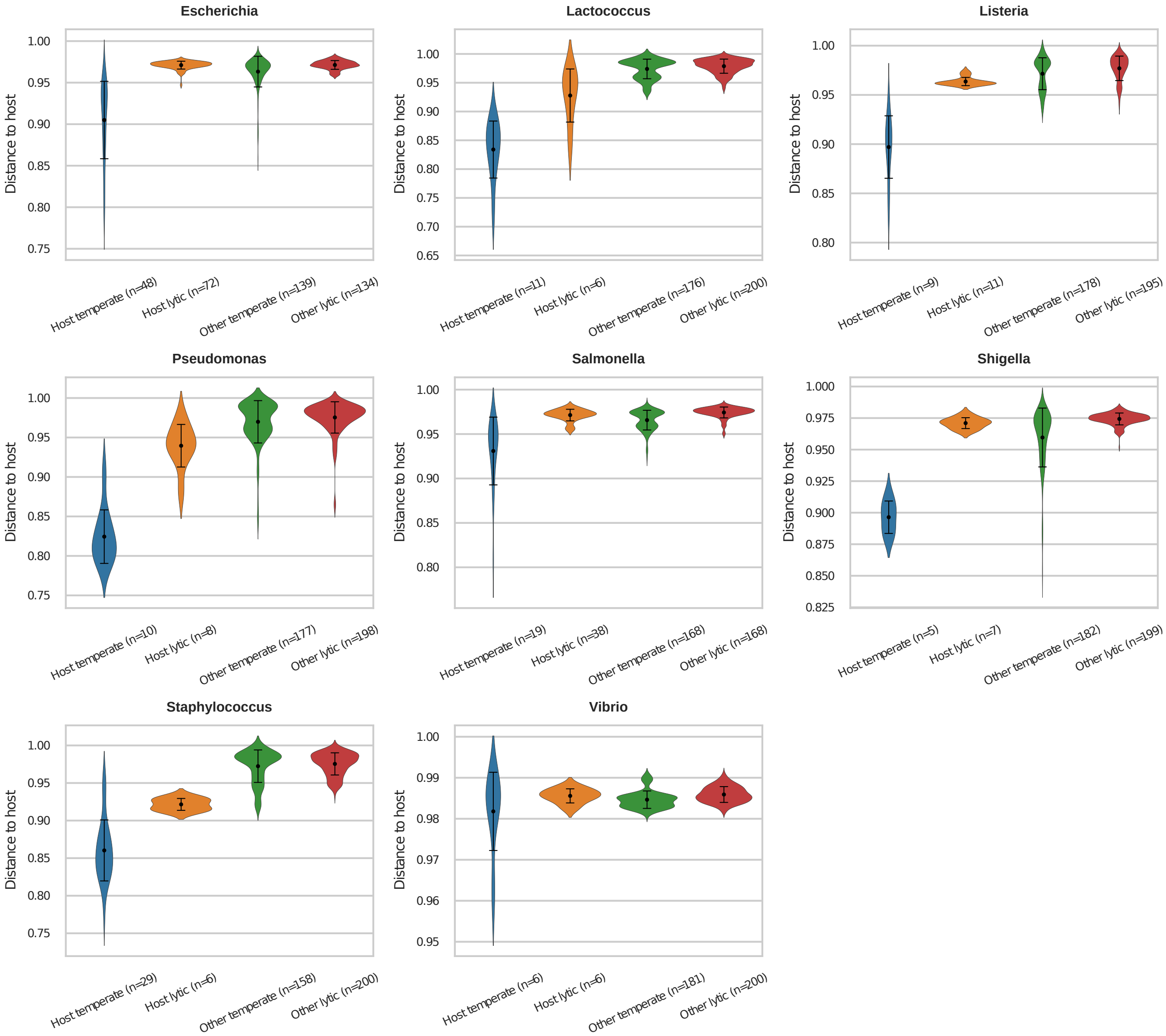

Supplementary Figure S6: Distances of 75 *Klebsiella* strains to 41 *Klebsiella* phages. Blue points indicate that no lytic interaction was observed in the laboratory. Panel a) shows k4freq- and panel b) k14exact-based distances.

a

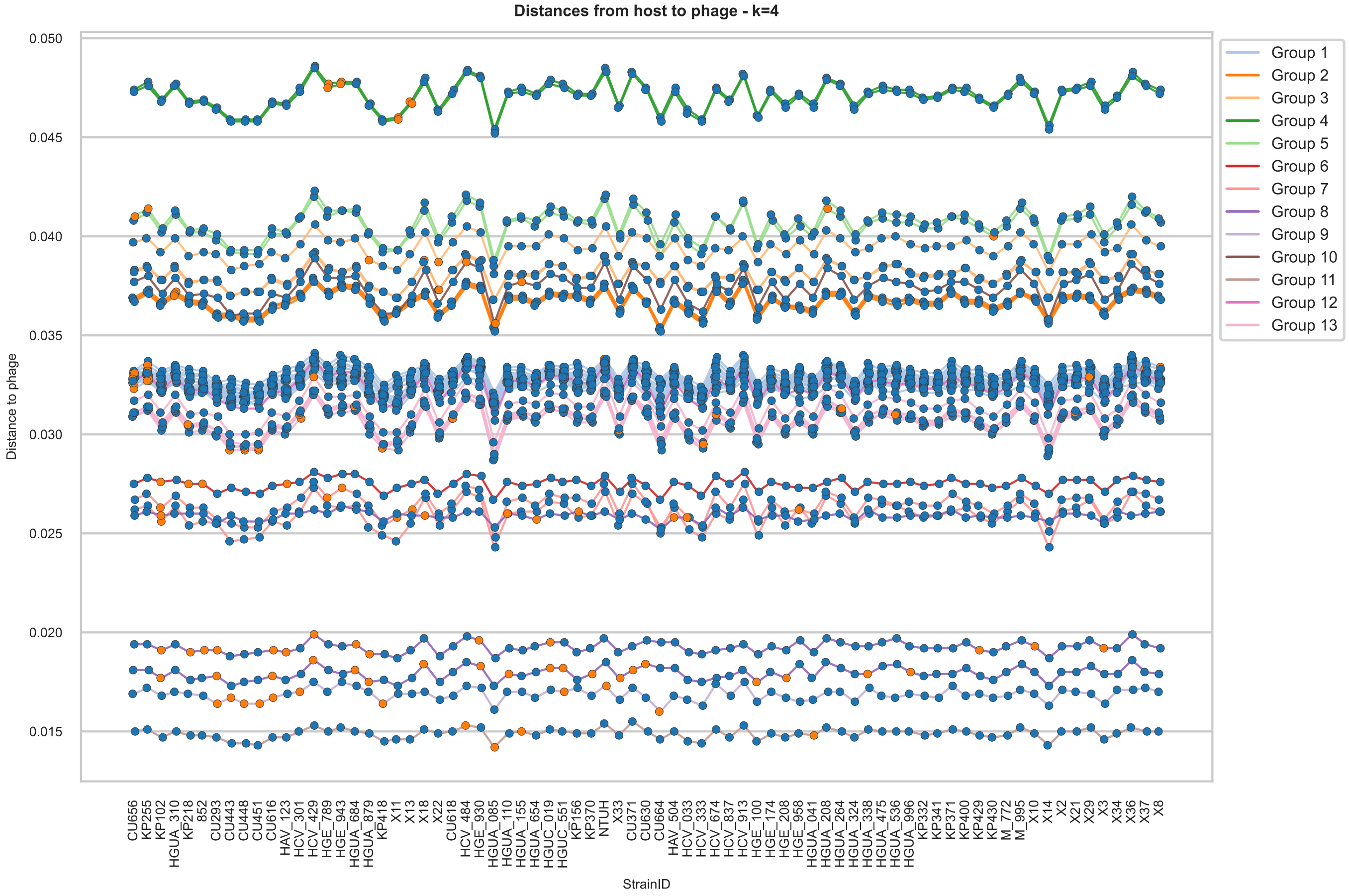

b

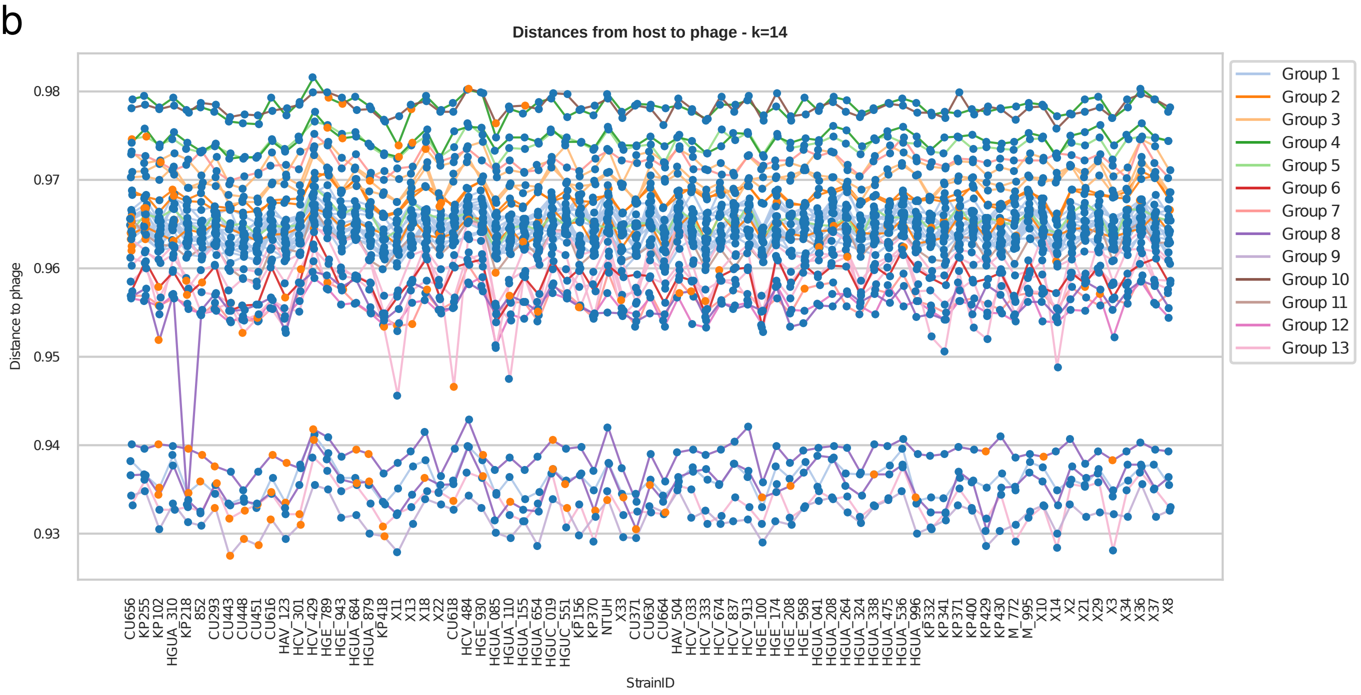

Supplement: Supplementary file 1 [file viruses-15-01196-s001.zip › SupplementaryFigures.pdf]
